# What’s in a name? Revisiting medicinal and religious plants at an Amazonian market

**DOI:** 10.1186/s13002-021-00433-4

**Published:** 2021-02-05

**Authors:** Isabela Pombo Geertsma, Mariana Françozo, Tinde van Andel, Mireia Alcántara Rodríguez

**Affiliations:** 1 Faculty of Science, University of Amsterdam, Science Park 904, Amsterdam, 1098 XH the Netherlands; 2 Faculty of Archaeology, Leiden University, Einsteinweg 2, Leiden, 2333 CC the Netherlands; 3 PI ERC BRASILIAE project, Faculty of Archaeology, Leiden University, Leiden, the Netherlands; 4 Clusius chair in History of Botany and Gardens, IBL, Leiden University, Leiden, the Netherlands; 5 Naturalis Biodiversity Center, PO Box 9517, Leiden, 2300 RA the Netherlands; 6 Biosystematics Group, Wageningen University, Wageningen, 6708 PB the Netherlands

**Keywords:** Afro-Brazilian religion, Medicinal plants, Ritual plants, Market survey, Ethnobotany, Candomblé, Tupi, Brazil, Vernacular names, Ver-o-Peso, Amazonia

## Abstract

**Background:**

In spite of an increasing number of ethnobotanical market surveys in the past decades, few studies compare changes in plant species trade over time. The open-air market Ver-o-Peso (VOP) in Belém, located near the mouth of the Amazon River in the state of Pará, Brazil, is known for its wide variety of medicinal plants. A survey of VOP was published in 1984, but it remains unknown to what extent its botanical composition changed over 34 years. Furthermore, in northern Brazil, little attention has been given to the origins of the vernacular names of these plants. Our aim is to give an up-to-date overview of the VOP medicinal plant market, concentrating on changes in species composition and vernacular names over time.

**Methods:**

We collected medicinal plants and vernacular names at VOP in August 2018. We identified most plants at the Museo Paraense Emilio Goeldi Herbarium, where we also deposited vouchers and specimen labels. We compared our species composition data to the 1984 inventory by Van den Berg. Furthermore, we investigated the etymologies of the vernacular plant names.

**Results:**

We recorded 155 plant specimens and 165 corresponding vernacular names, and collected 146 specimens from the medicinal and ritual stalls of VOP reporting 86 species formerly not recorded at this market. Vernacular names had mostly Portuguese roots, followed by Tupi and African ones. We found 30 species also documented in 1984, and vernacular names that overlapped between both surveys were used for the same botanical species or genus, indicating that vernacular names have changed little in the past decades. Lastly, we found 26 more introduced species sold at VOP compared to 1984.

**Conclusions:**

Forest degradation and deforestation, prevalence of diseases, and methodological factors may play a role in the differences we found in our survey compared to 1984. Of the plants that did overlap between the two surveys, vernacular names of these plants were hardly different. Lastly, the lingual origins of the vernacular names in our survey and the origins of the plant species reflect the history of the intricate syncretism of medicinal plant practices of indigenous, Afro-Brazilian and European origins in Belém.

**Supplementary Information:**

The online version contains supplementary material available at 10.1186/s13002-021-00433-4.

## Background

Many people around the world rely on traditional healthcare systems involving medicinal plants, which are often sold at open-air markets [1–5]. This is also the case in Brazil, where a great variety of medicinal plants are still traded at open-air markets [6–8]. These markets are hosted and visited by Brazilians and tourists from various cultural backgrounds. This cultural diversity is reflected in the number of available plant species and their uses [9]. It is easy to establish which medicinal plants are popular in the region through market surveys, as markets give an overview of local demand [10].

There has been an increasing number of ethnobotanical market surveys in the past decades [11]. Although there are a number of studies that compare different markets to each other in terms of availability of plant species [12–14], few studies have repeated ethnobotanical surveys in markets that were already monitored decades before to examine changes in species composition [15, 16]. Likewise, few market surveys have included an analysis of vernacular names of medicinal species being sold [12, 17]. The availability of plant products at markets changes over time [16], indicating the importance of periodically conducting market surveys for a more complete picture of plant availability and to assess possible impacts of social and environmental factors [15, 18, 19]. Furthermore, the analysis of vernacular names associated with plant species can inform us more about people’s cultural and botanical history in the study area [17].

The famous open-air market Ver-o-Peso (VOP) is located in Belém, near the mouth of the Amazon River in the state of Pará, Brazil. This popular market functions as a source of medicinal plants for nearby smaller markets and is known for its wide variety of herbal medicine [20, 21]. In 1984, Van den Berg [1] published the only ethnobotanical survey of the VOP, listing the most common plants encountered in each section of the market (medicinal and ritual plants, handicrafts, vegetables and root crops, fruits, horticultural and ornamentals). This study provided an overview of the most popular species; however, this approach possibly underestimated the total medicinal species composition of the market. Furthermore, it remains unknown to what extent the botanical composition of this major Amazonian market has changed over the past 34 years.

The aim of this paper was to compile an up-to-date botanical overview of the VOP medicinal plant market, concentrating on species composition and vernacular names. Specifically, we wanted to know what plant species are sold at the medicinal stalls of VOP today and how these differed from those listed by Van den Berg [1]. We also compared the vernacular names for species in use today with those reported by Van den Berg [1]. Furthermore, we investigated whether the origin of currently marketed plant species and their attributed vernacular names reflected the cultural backgrounds of the VOP sellers. Our three hypotheses were as follows. First, we expected to find a higher and different medicinal species composition due to our methodological approach, which differed from that of previous studies [1, 15], and because the diversity of medicinal plants being offered for sale changes over time [16]. Availability of plant species changes from time period to time period, certain plants are conserved, new species are added, and other species are lost over time [16]. Second, we hypothesize that vernacular plant names would remain similar over time, as previously found in the Amazonian context [14, 22]. Thirdly, with their own language backgrounds, religious faiths, and practices, the VOP sellers are of mixed descent: indigenous, African, and European [23]. This mix of peoples is found throughout Brazil and influences the Brazilian Portuguese lexicon, including names of flora and fauna [24]. Afro-Brazilians of the Bahia state in northeastern Brazil, where a considerable number of enslaved Africans were forcefully brought to, sometimes use African-derived names for plants used in a medicinal and ritual context [25]. Also, many plant names in Surinam, a neighboring country to Brazil with a comparable history in the triangular trade involving the dislocation of western Africans, have African etymologies, influenced by the enslaved peoples that were forced to work there under brutal conditions [26, 27]. Therefore, following these examples, we hypothesized that the multicultural origins of the current population of north Brazil, including VOP sellers and consumers, would be reflected in the species’ origin and vernacular names [23, 28]. In the end, the outcome of this research contributes to the understanding of the present biocultural diversity of one of the largest and most biodiverse markets in South America.

## Methods

### Study area: Ver-o-Peso market

**Fig. 1:**
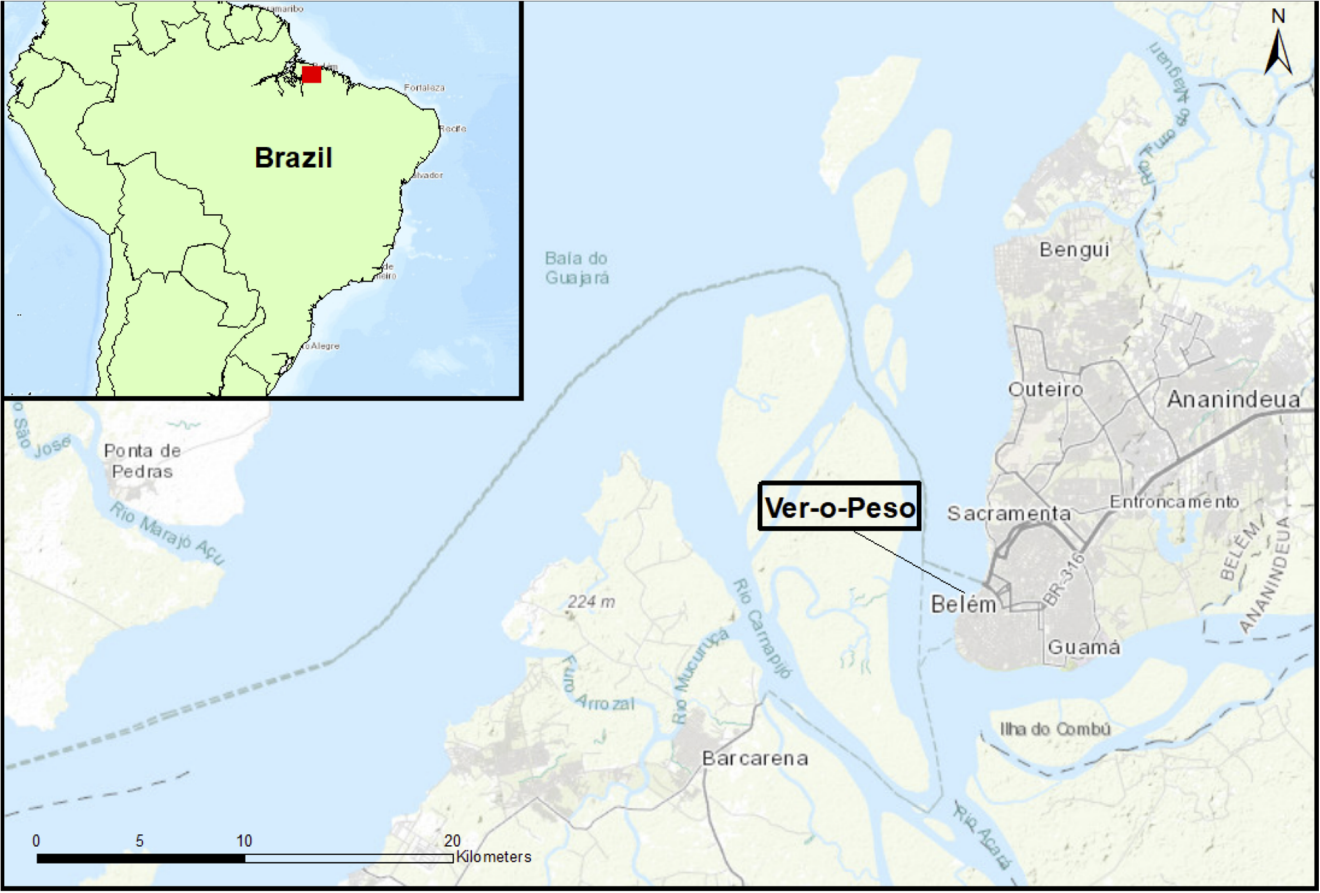
Map of the study area. Red square in the upper left map indicates Belém’s location; larger map shows the location of the VOP market on the banks of the Pará river

**Fig. 2:**
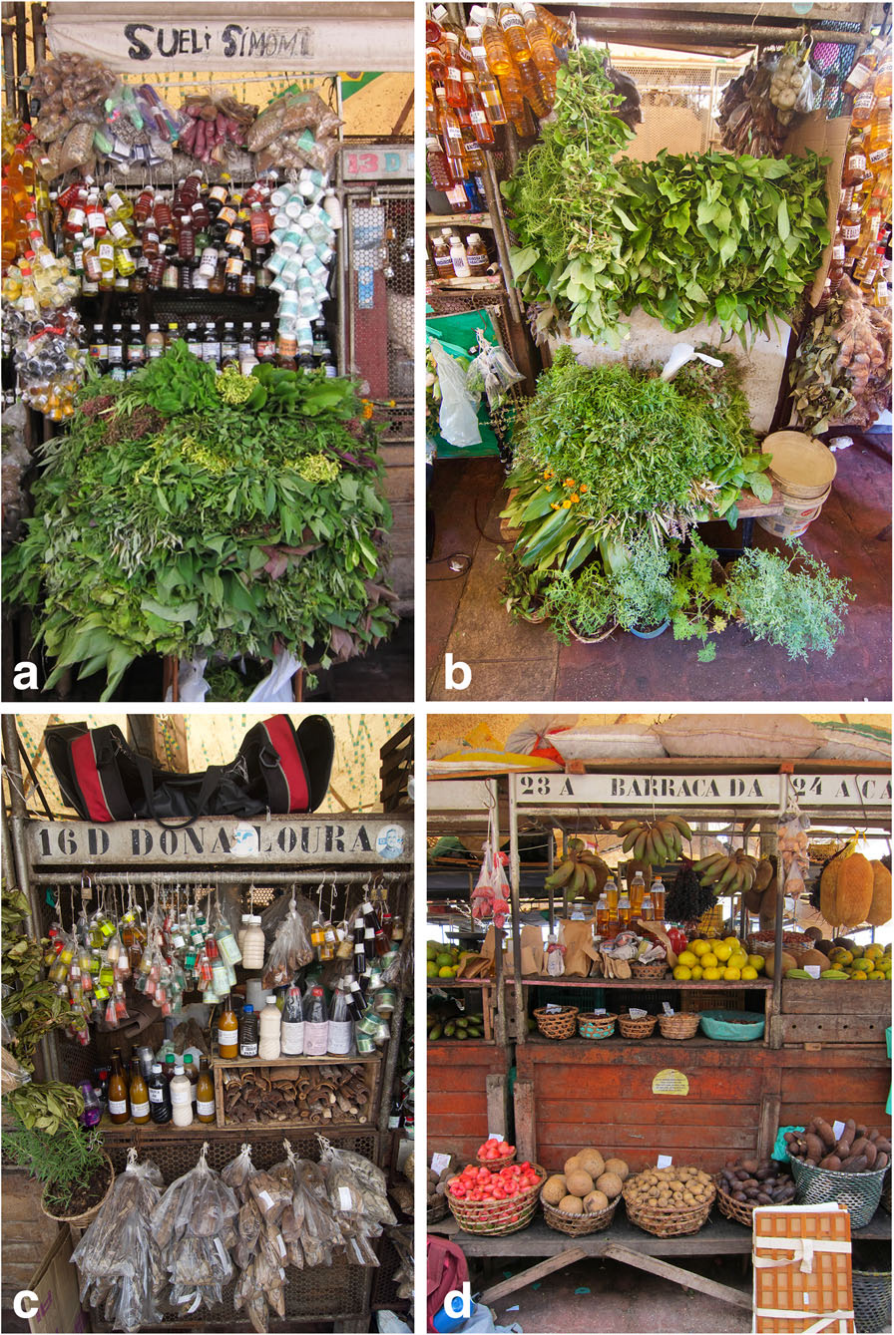
Market stalls at VOP. **a**, **b** Stalls selling fresh medicinal plants. **c** Stall that sells barks in plastic bags. d Fruit and seed stall. Photos by I. Pombo Geertsma (a and d) and C.A. van der Hoeven (b and c)

The VOP is situated on the bank of the river Pará, a tributary to the Amazon River, in the city of Belém (Figs. 1 and 2). It is a daily open market that caters to a large and varied public and offers several types of products, from fish to artisanal craftwork, which are offered for sale in separate areas [1, 23, 29]. We collected the medicinal plants at the VOP between the 7th and 25th of August of 2018 during ten mornings between 6:30 and 10:00 am. We checked the stalls for the freshest plants and bought a varying number of plants on each visit. Prior to buying the specimens, we introduced ourselves in Portuguese to the vendors, explained the framework of this research project, and highlighted that we were collecting medicinal and ritual plant specimens for the Herbarium of the Museu Paraense Emilio Goeldi (MG). We followed the ISE Code of Ethics [30]. On the 25th of August, we counted 80 medicinal plant stalls and 50 *erveiras* and *erveiros*, female and male vendors respectively.

### Plant collection and identification

We collected plants at the medicinal stands and at one artisanal stand known for its selection of dry fruits and seeds with medicinal applications. Popular plants that we could accurately identify on the spot, such as *Allium cepa* L. (onion) and *Ruta graveolens* L. (arruda), were not collected. We obtained oral informed consent from the vendors before buying the plants in the units in which they were sold. We documented vernacular names, date, price, and vendor’s names in a notebook. We asked for the vernacular names to be repeated several times for the correct notation. Following Albuquerque et al. [10], we made photographs of the specimens and close-up pictures of every plant in several angles, and we pressed the plants in a standard plant press to make herbarium vouchers. We either dried the specimens at home with a hairdryer, blowing hot air into a plastic bag opened at the end, in which the plant press was placed; or in the drying stove of the MG (TE-394/4, at 70 °C circulation and air renewal turned on). To disinfect our collections, we stored the dried plants in a freezer provided by the MG. After identification, we deposited all vouchers and specimen labels at the MG to become part of their collection and coded each plant according to the abbreviation of the first author: IPG.

We identified most plants by comparing them to herbarium vouchers at the MG and in the field during informal walks in nearby areas where these plants were presumably collected, such as at Ilha do Combu and the Parque Estadual do Utinga. Photographs of those specimens that we could not identify at the MG were compared to South American collections at the Herbarium of Naturalis Biodiversity Center (L) in Leiden, the Netherlands. We completed our identifications by consulting the Global Biodiversity Information Facility (GBIF) website [31], the online checklist of the Flora do Brasil 2020 [32], the Tropicos database [33], and literature on medicinal plants in Brazil [34, 35] and Suriname [36]. We identified seeds and barks by comparing their vernacular names with those mentioned in literature on Brazilian useful plants [21, 35, 37–41], and comparing them with photographs in Google Images. We checked and updated the species scientific plant names by using The Plant List [42].

### Data analysis

We organized the following information for each recorded specimen in an Excel sheet: family, genus, species and collection number, vernacular name, language, plant part sold, status in Brazil (native or introduced). We verified the spelling of the names, first with the help of the vendors in the market, and later by using Corriente [43], Navarro [44], DATAPLAMT [45], Dicionário ilustrado Tupi Guarani [46], and Michaelis Dicionário Brasileiro da Língua Portuguesa [47]. The Tupi and Portuguese dictionaries, along with the Online Etymology Dictionary [48], Corriente [43], Sebba and Corbacho [49], and Fernandes and Soares [50] were used to determine the etymology of the vernacular plant names. Names with Latin, Greek, Phoenician (in the case of Malaga), and Arabic etymologies were listed as Portuguese, as these words were implemented into Portuguese language prior to entering Brazilian Portuguese vocabulary [24, 51].

To check the phytogeographical distribution, its status in Brazil, and the origin of each plant species, we used the Tropical Plant Database [52], Missouri Botanical Garden Plant Finder [53], GBIF [31], PROTA4A [54], PROSEA [55], Van Andel et al. [56], the Flora do Brasil 2020 online checklist [32], the Flora of China [57], Kew Science Plants of the World online [58], the Catalogue of Life Annual Checklist [59], Tropicos [33], the Naturalis Bioportal [60], and Herbarium voucher labels at MG. To compare our results to the VOP ethnobotanical inventory in the 1980s, we updated the taxonomy of the plant species found at the medicinal and religious plant stalls by Van den Berg [1] and checked if they were native or introduced. Comparing detailed medicinal or ritual uses of plants between 1984 and 2018 was not part of our research aims.

### Data sharing

This study was based on the participation of local specialist vendors. Although we compensated them financially by buying their plants, we also discussed our research results with them and followed up their request to provide them with a complete list of the common and scientific names of the identified plants, following Del Arco et al. [61]. We also included pictures of some pressed and dried vouchers, our contact information, and the MG location. We decided that this was the best option due to time constraints preventing us from organizing workshops or other benefit-sharing activities.

## Results

### Inventory of medicinal and ritual plants at the VOP in 2018

**Fig. 3:**
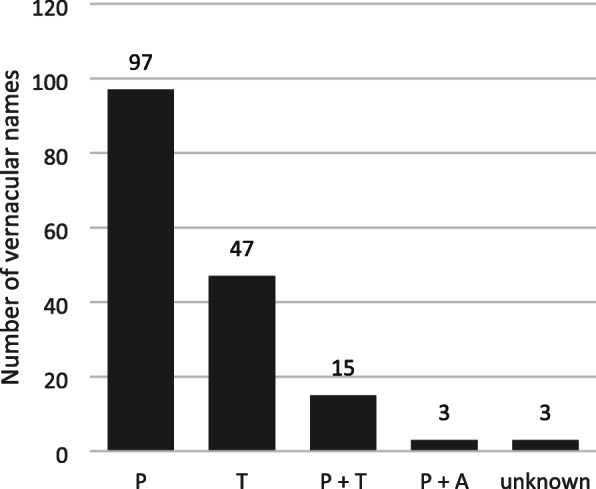
Linguistic origin of the vernacular plant names of the Ver-o-Peso market, 2018. Languages are Portuguese (P), Tupi (T), and African (A)

We recorded 155 plant specimens with in total 165 corresponding vernacular names and collected 146 specimens from the medicinal stalls of the VOP (Table 1). Most of the vernacular names had Portuguese roots (59%), followed by Tupi (28%) roots, while three names had unknown origins (2%) (Fig. 3). If a name had a double etymological root, they were classified in separate categories. We found names that had a combination of Tupi and Portuguese roots (9%) and African and Portuguese roots (2%). For example, *uxi-amarelo* (*Endopleura uchi* (Huber) Cuatrec.) is a combination of a Tupi plant (*uxi*) and the Portuguese term for yellow (*amarelo*). Likewise, *Tapete de Oxalá* (*Episcia cupreata* (Hook.) Hanst.) is a combination of an African term (the god Oxalá) and the Portuguese term for rug.

**Table 1:** Medicinal plant species documented at VOP

Family, scientific name/voucher number	Vernacular name/language	Part sold
Acanthaceae		
cf. *Blechum* sp./IPG20	Amansa/P	Fresh aerial parts
*Justicia pectoralis* Jacq./IPG17, IPG44	Abre caminho/P	Fresh aerial parts
Adoxaceae		
*Sambucus canadensis* L./IPG24, IPG57	Sabugueiro/P	Branches, fresh leaves, flowers
Amaranthaceae		
*Alternanthera brasiliana* (L.) Kuntze/IPG6, IPG78	Chega até a mim/P, Meracilina/?	Fresh aerial parts
*Pfaffia glomerata* (Spreng.) Pedersen/IPG33	Corrente/P	Fresh aerial parts
Amaryllidaceae		
*Allium cepa* L./not collected	Cebola/P	Bulb’s skin
Anacardiaceae		
*Anacardium* cf. *giganteum* Hancock ex Engl./IPG100	Caju-í do mato/T + P	Bark
*Anacardium occidentale* L./IPG99	Cajú/T	Bark
*Antrocaryon amazonicum* (Ducke) B.L. Burtt & A.W. Hill/IPG103	Cedro/P	Bark
*Schinus terebinthifolia* Raddi/IPG14	Aroeira/P	Branches, fresh leaves, flowers
*Spondias* sp./IPG130	Taperebá/T, Cajá/T	Bark
Anacardiaceae sp. IPG94	Anoera/T?	Bark
Anacardiaceae sp. IPG95	Aroeira/P	Bark
Annonaceae		
*Annona montana* Macfad./IPG81	Graviola/P	Branches, fresh leaves
*Xylopia frutescens* Aubl./IPG155	Ibiriba/T	Dry fruit and seed
Annonaceae sp./IPG107	Cipó urira/T	Woody stem with
Apocynaceae		
*Aspidosperma nitidum* Benth. ex Müll.Arg./IPG101	Carapanauba/T	Bark
*Cascabela* cf. *thevetia* (L.) Lippold/IPG148	Castanha da India/P, Munduruku/T, Chapeu de mato leão/P	Dry fruit and seed
*Himatanthus articulatus* (Vahl) Woodson/IPG129	Sucuúba/T	Bark
Araceae		
*Dieffenbachia seguine* (Jacq.) Schott/IPG79	Comigo-ninguém-pode/P	Fresh aerial parts
Araliaceae		
*Polyscias scutellaria* (Burm.f.) Fosberg/IPG73	Cuia mansa/T + P	Fresh aerial parts
Arecaceae		
*Manicaria saccifera* Gaertn./IPG144	Buçu/T	Fruit and seeds
*Socratea exorrhiza* (Mart.) H.Wendl./IPG124	Paxiúba/T	Root
Asparagaceae		
*Sansevieria cylindrica* Bojer ex Hook./not collected	Lança-de-São Jorge/P	Whole plant
*Sansevieria hyacinthoides* (L.) Druce/not collected	Espada-de-são Jorge/P	Whole plant
*Sansevieria trifasciata* Prain/not collected	Espada-de-Joana d’Arc/P	Whole plant
Bignoniaceae		
*Bignonia* cf. *nocturna* (Barb.Rodr.) L.G.Lohmann/IPG104	Cipó curimbó/T	Woody stem with bark
*Fridericia* cf. *chica* (Bonpl.) L.G.Lohmann/IPG77	Pariri/T	Fresh aerial parts
*Mansoa alliacea* (Lam.) A.H.Gentry/IPG60	Cipó de Alho/T + P	Fresh aerial parts
*Newbouldia laevis* (P.Beauv.) Seem./IPG82	Espinheira santa/P	Dry leaves
Bixaceae		
*Bixa orellana* L./IPG174	Urucú/T	Fruit and seeds
Boraginaceae		
*Cordia* sp./IPG115	Louro rosa/P	Wood with bark
Burseraceae		
*Protium* sp./IPG97	Breu/P	Bark
Clusiaceae		
*Calophyllum brasiliense* Cambess./IPG111	Jacareuba/T	Bark
*Symphonia globulifera* L.f./IPG92	Anani/T	Bark
Commelinaceae		
*Commelina erecta* L./IPG31	Vence-demanda/P	Fresh aerial parts
*Tradescantia zebrina* Bosse/IPG85	Quebra-chibança/P, Trapoeraba/T	Fresh aerial parts
Compositae		
*Acmella oleracea* (L.) R.K.Jansen/IPG1	Jambu/T	Fresh whole plant
*Ayapana triplinervis* (Vahl) R.M.King & H.Rob/IPG55	Japana branca/T + P	Fresh aerial parts
*Bidens* sp./IPG18	Picão/P	Fresh whole plant
*Mikania glomerata* Spreng./IPG54, IPG75	Sicuriju/T	Fresh aerial parts
*Pectis elongata* Kunth/IPG9	Cominho/P	Fresh aerial parts
*Sphagneticola trilobata* (L.) Pruski/IPG16	Desempata/P	Fresh whole plant
*Tagetes erecta* L./IPG12	Cravo/P	Fresh aerial parts
*Unxia camphorata* L.f./IPG51	Trevo são João/P	Fresh whole plant
Convolvulaceae		
*Cuscuta* sp./IPG65	Desatrapalha/P	Fresh whole plant
Costaceae		
*Costus spicatus* (Jacq.) Sw./IPG59	Canarana/P + T	Fresh aerial parts
Crassulaceae		
*Bryophyllum pinnatum* (Lam.) Oken/IPG56	Pirarucu/T	Fresh aerial parts
Cucurbitaceae		
*Cayaponia* cf. *rigida* (Cogn.) Cogn./IPG63	Quebra feitiço/P	Fresh aerial parts
*Luffa operculata* (L.) Cogn./IPG149	Cabacinha/P	Dry fruit
*Momordica charantia* L./IPG15	Melão-de-São Caetano/P	Fresh aerial parts
Dilleniaceae		
*Doliocarpus dentatus* (Aubl.) Standl./IPG105	Cipó-de-fogo/P	Woody stem with bark
Dioscoreaceae		
*Dioscorea* sp./IPG176	Batata-de-colar-osso/P	Rhizome
Euphorbiaceae		
*Croton cajucara* Benth./IPG128	Sacacá/T	Bark
*Croton sacaquinha* Croizat/IPG5	Angel-de-guarda/P, Corre atraz/P, Busca longe/P	Fresh aerial parts
*Euphorbia tithymaloides* L./IPG76	Coramina/P	Fresh aerial parts
*Jatropha curcas* L./IPG47	Pião branco/P	Fresh aerial parts
*Jatropha gossypiifolia* L./IPG19	Pião roxo/P	Fresh aerial parts
*Jatropha podagrica* Hook./IPG38	Pião-paje/P + T	Fresh aerial parts
Geraniaceae		
*Pelargonium* cf. *graveolens* L’Hér/not collected	Malva rosa/P	Living plant
Gesneriaceae		
*Episcia cupreata* (Hook.) Hanst./IPG40	Tapete de Oxalá/P + A (Oxalá), Laço de amor/P	Fresh whole plant
Humiriaceae		
*Endopleura uchi* (Huber) Cuatrec./IPG134	Uxi-amarelo/T + P	Bark
*Humiria balsamifera* Aubl./IPG117, IPG132	Miri/T; Umiri/T	Bark
Lamiaceae		
*Aeollanthus suaveolens* Mart. ex Spreng./IPG11	Catinga-de-mulata/T + P	Fresh whole plant
*Ocimum americanum* L./IPG7, IPG8	Estorakue/P, Manjericão/P	Fresh aerial parts
*Ocimum basilicum* L./IPG62	Manjericão roxo/P	Fresh whole plant
*Ocimum gratissimum* L./IPG52	Alfavaca/P	Fresh aerial parts
*Plectranthus amboinicus* (Lour.) Spreng./IPG68	Hortelã-de-Maranjão/P + T, Hortelã-de-folha-grande/P	Branch with fresh leaves
*Pogostemon cablin* (Blanco) Benth./IPG86	Anica/P	Fresh aerial parts
*Pogostemon heyneanus* Bent./IPG58	Oriza/P	Fresh aerial parts
*Vitex agnus-castus* L./IPG4	Alecrim-de-Angola/P (alecrim) + A (Angola)	Fresh aerial parts
Lamiaceae sp. IPG61	Chama/P	Fresh aerial parts
Lauraceae		
*Cinnamomum verum* J.Presl/IPG120	Canela/P	Woody stem with bark
*Cinnamomum* sp./IPG46	Canela/P	Fresh aerial parts
Lecythidaceae		
*Couratari guianensis* Aubl./IPG131	Tauari/T	Bark, fruits
Leguminosae		
*Anadenanthera* cf. *peregrina* (L.) Speg./IPG121	Paricá/T	Bark
*Bauhinia* cf. *guianensis* Aubl./IPG110	Escada-de-Jabotí/P + T	Woody stem with bark
*Bauhinia monandra* Kurz/IPG72	Pata-de-vaca/P	Fresh aerial parts
*Bowdichia virgilioides* Kunth/IPG172	Sucupira/T	Seeds
*Caesalpinia ferrea* C.Mart./IPG158	Jucá/T	Fruit
*Copaifera* sp./IPG108	Copaíba/T	Bark
*Dalbergia monetaria* L.f./IPG135	Verônica/P	Woody stem with bark
*Dipteryx odorata* (Aubl.) Willd./IPG152	Cumaru/T	Seeds
*Hymenaea courbaril* L./IPG112	Jatobá/T	Bark
*Mimosa tenuiflora* (Willd.) Poir./IPG114	Jurema preta/T + P	Bark
*Mimosa verrucosa* Benth./IPG113	Jurema branca/T + P	Bark
cf. *Ormosia* sp./IPG98	Buiuçú/T	Bark
*Pentaclethra* sp./IPG125	Pracaxí/T	Bark
*Senna hirsuta* (L.) H.S.Irwin & Barneby/IPG50	Sombra-do-mundo/P, Afasta espirito/P	Fresh aerial parts
*Vouacapoua americana* Aubl./IPG89	Acapú/T	Wood
Leguminosae sp./IPG96	Barbatimão/T	Bark
Lythraceae		
*Punica granatum* L./IPG169	Romã/P	Dry exocarp
Malvaceae		
*Gossypium barbadense* L./IPG42	Algodão/P	Fresh aerial parts
*Luehea* sp./IPG90	Açoita-cavalo/P	Bark
Melastomataceae		
*Miconia ciliata* (Rich.) DC./IPG26	Canela-de-velho/P	Fresh aerial parts
Meliaceae		
*Carapa guianensis* Aubl./IPG93	Andiroba/T	Bark
Moraceae		
*Brosimum acutifolium* Huber/IPG118	Mururé/T	Bark
*Dorstenia cayapia* subsp. *asaroides* (Hook.) C.C. Berg/IPG48	Apií/T	Fresh whole plant
*Morus nigra* L./IPG28	Amora/P	Branch with fresh leaves and fruits
Olacaceae		
*Ptychopetalum olacoides* Benth./IPG116	Marapuama/T	Wood with bark
Phyllanthaceae		
*Phyllanthus amarus* Schumach. & Thonn./IPG22	Quebra-pedra/P	Fresh whole plant
*Phyllanthus urinaria* L./IPG21	Dinheiro-em-penca/P	Fresh aerial parts
Phytolaccaceae		
*Petiveria alliacea* L./IPG25, IPG179	Mucuraca-á/T, Rinchão/P	Fresh aerial parts; Dry whole plant
Piperaceae		
*Peperomia circinnata* Link/IPG64	Carrapatinha/P	Fresh aerial parts
*Piper callosum* Ruiz & Pav./IPG27	Elixir-paregórico/P	Fresh aerial parts
*Piper peltatum* L./IPG70	Malvarisco-de-folha-grande/P, Capeba/T	Fresh leaves
Plantaginaceae		
*Bacopa monnierioides* (Cham.) B.L.Rob./IPG84	Trevo do mar/P	Fresh aerial parts
*Conobea scoparioides* (Cham. & Schltdl.) Benth./IPG32	Pataqueira/P	Fresh aerial parts
*Scoparia dulcis* L./IPG13	Vassourinha-de-igreja/P	Fresh whole plant
Poaceae		
*Chrysopogon zizanioides* (L.) Roberty/IPG178	Patichuli/P	Dry roots
*Cymbopogon citratus* (DC.) Stapf/not collected	Capim-marinho/T + P	-
*Zea mays* L./IPG164	Milho/P	Stalk
Polygonaceae		
*Antigonon leptopus* Hook. & Arn./IPG69	Agarradinho/P	Whole plant with tubers
*Polygala spectabilis* DC./IPG36	Camembeca/T	Fresh whole plant
Portulacaceae		
*Portulaca pilosa* L./IPG23	Amor crescido/P	Fresh whole plant
Rhizophoraceae		
*Rhizophora mangle* L./IPG126	Raíz-de-mangue/P	Root
Rosaceae		
cf. *Prunus* sp./IPG91	Ameixa/P	Bark
Rubiaceae		
*Uncaria* sp./IPG133	Unha-de-gato/P	Woody stem with bark
Rutaceae		
*Citrus* x *aurantium* L./IPG67	Laranja-da-terra/P	Branch with fresh leaves
*Ruta graveolens* L./not collected	Arruda/P	Leaves in small plastic bags; Whole plant
Sapindaceae		
*Paullinia cupana* Kunth/IPG154	Guaraná/T	Seeds
Sapotaceae		
*Pradosia lactescens* (Vell.) Radlk./IPG102	Casca doce/P	Bark
Selaginellaceae		
*Selaginella parkeri* (Hook. & Grev.) Spring/IPG49	Samambaia/T	Fresh whole plant
Simaroubaceae		
*Quassia amara* L./IPG41, IPG123	Folha-da-quina/P; Pau-tenente/P	Fresh aerial parts; Wood with bark
Siparunaceae		
*Siparuna guianensis* Aubl./IPG88	Capitiú/T, Negra-mina/P	Fresh aerial parts
Solanaceae		
*Capsicum annuum* L./IPG29	Pimenta malagueta/P	Fresh aerial parts
*Physalis angulata* L./IPG37	Camapú/T	Fresh whole plant
Urticaceae		
*Cecropia obtusa* Trécul/IPG87	Imbaúba branca/T + P	Dry leaves
*Pellionia repens* (Lour.) Merr./IPG71	Hei-de-vencer/P, Vence-batalha/P, Maria-fumaça/P	Fresh whole plant
Verbenaceae		
*Aloysia gratissima* (Gillies & Hook.) Tronc./IPG39	Folha-de-alfazema/P	Fresh aerial parts
*Lippia alba* (Mill.) N.E.Br. ex Britton & P.Wilson/IPG43, IPG66	(Erva) cidreira/P	Fresh aerial parts
*Lippia thymoides* Mart. & Schauer/IPG30	Manjerona-de-Angola/P + A	Fresh aerial parts
Vitaceae		
*Cissus verticillata* (L.) Nicolson & C.E.Jarvis/IPG10, IPG34, IPG80	Insulina/P, Cipó-de-puca/T, Quebra-barreira/P	Fresh aerial parts
Xanthorrhoeaceae		
*Aloe vera* (L.) Burm.f./not collected	Babosa/P	Whole plant
Zingiberaceae		
*Alpinia zerumbet* (Pers.) B.L.Burtt & R.M.Sm./IPG45	Vindicá/P	Fresh aerial parts
*Curcuma longa* L./not collected	Mangarataia/T, Safrão/P, Açafria/P, Gengibre amarela/P	Rhizome
Unidentified		
Indet/IPG74	Chora-nos-meus-pés/P	Fresh aerial parts
Indet/IPG83	Cabi/uncertain	Fresh aerial parts
Indet/IPG106	Cipó-ferro/T + P	Bark
Indet/IPG109	Core/P	Bark
Indet/IPG122	Pau-de-bota/P	Woody stem with bark
Indet/IPG127	Raiz-do-sol/P	Root
Indet/IPG177	Cauan/T	Fresh tuber

Fresh aerial parts include stem, leaves, flowers, and/or fruit

*Indet* species indetermined; languages: *P* Portuguese, *T* Tupi, *A* African

Most of our recorded specimens consisted of fresh plants, followed by woody stems or barks, and a few fruits, seeds, roots/rhizomes, and dried herbs (Table 2).

**Table 2:** Plant parts sold at medicinal and religious stalls at the VOP market

Plant parts	Number of medicinal plant species
Whole plants (fresh)	82
Woody stems and/or barks	44
Fruits	9
Whole plants (dried)	8
Seeds	7
Roots/rhizomes	6

Of these, we identified 116 taxa to species level, 14 taxa to genus level, five to family level, and seven taxa remained unidentified. Species were spread over 59 families, the most diverse family at the market was Leguminosae (12% of the species), followed by Lamiaceae (7%), Compositae (6%), Euphorbiaceae (4%), Anacardiaceae, and Bignoniaceae (3%) (Table 3). We found 77 (66%) species native to Brazil and 39 (34%) previously introduced from Africa, Asia, Europe, and other Latin-American countries (see Additional file 1). We documented 11 species of African origin, 14 species of Asian origin, and three species of European origin. All African and almost all Asian species had a tropical distribution, except *Allium cepa* L. [54], *Morus nigra* L. [62], and *Punica granatum* L. [63], which originated in the drier parts of southwestern Asia.

**Table 3:** Number of species per medicinal plant family at the VOP market

Plant families	Number of medicinal plant species
Leguminosae	16
Lamiaceae	9
Compositae	8
Euphorbiaceae	6
Anacardiaceae	4
Bignoniaceae	4
Other^a^	88

^a^This category contains 53 families with less than four species

### Comparison between current VOP medicinal plants and VOP in the 1980s

**Fig. 4:**
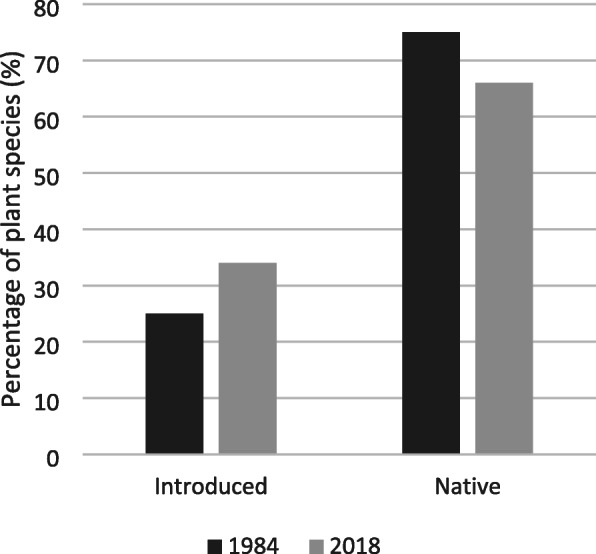
Comparison of the proportion of native versus introduced species between Van den Berg’s (1984) survey and our inventory (2018)

**Fig. 5:**
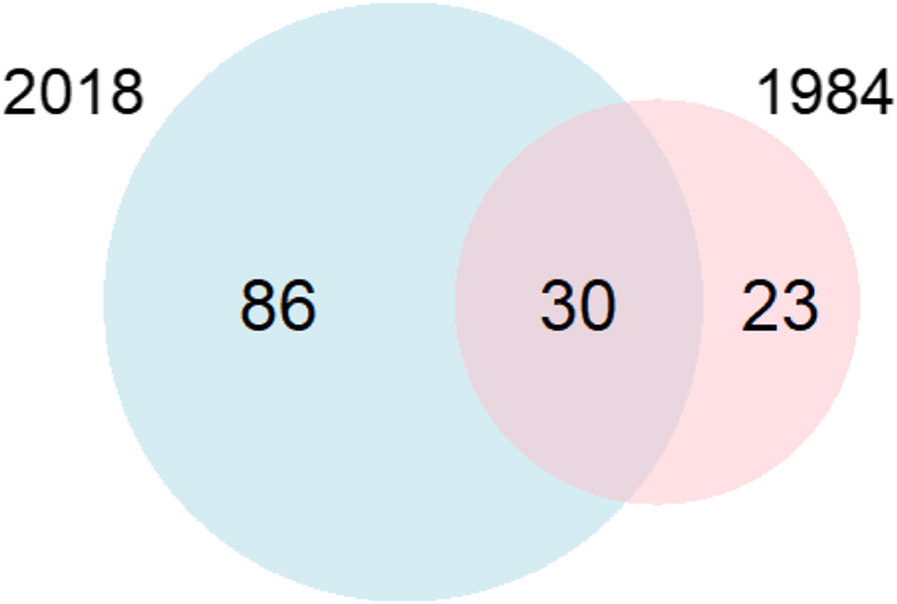
Venn-diagram showing the overlap between 2018 and the period 1965–1984 [1] of plant species found at VOP’s medicinal and ritual stalls

We compared our survey to Van den Berg’s medicinal and religious plant survey in 1984 [1]. Van den Berg [1] listed 39 (75%) native and 13 (25%) introduced species (Fig. 4). In our survey, we found 30 species (57%) that were also documented in 1984 [1] (Fig. 5). For these overlapping species, we documented three vernacular names not mentioned by Van den Berg [1]. Likewise, she documented four vernacular names that did not come up in our survey (Table 4). Overlapping vernacular names were used for the same botanical species or genus.

**Table 4:** Differences in vernacular names found in the current study and in Van den Berg [1]

Species	Current vernacular names	Vernacular names reported by Van den Berg [1]
*Cissus verticillata*	Cipó de Puca, Insulina, Quebra barreira	Pucá
*Fridericia* cf. *chica*	Pariri	Pariri; Crajirú
*Petiveria alliacea*	Mucuraca-á; Rinchão	Mucura-caá; Guiné
*Piper callosum*	Elixir paregórico	Elixir paregórico; Óleo elétrico
*Bryophyllum pinnatum*	Pirarucu	Pirarucu; Folha-da-fortuna

## Discussion

### Medicinal plants at the Ver-o-Peso

Our results give an up-to-date botanical inventory of the VOP, one of the largest open markets in South America, which is often quoted in ethnobotanical review studies [11, 14, 18]. Likewise, we were able to complement other medicinal plant market surveys in the area that differ in species composition and richness [8, 19, 20], providing a more accurate idea of the present diversity of medicinal plants in the area. Although subject of a previous ethnobotanical study [1], comparison with our intensive market inventory resulted in differing degrees of overlap in species assembly and nomenclature.

The differences in the species inventory we found compared to Van den Berg [1] may be caused by social or medical factors, such as the incidence of certain diseases for which our newly recorded plants are used for healing. For example, the prevalence of diabetes in Brazilian adults has increased since the 1980’s [64], and both *Momordica charantia* and *Bauhinia* species are employed to treat it in Brazil [6, 16]. *Bauhinia* species are also used to treat high cholesterol, kidney diseases, and high blood pressure [15, 16]. Although recovered by our survey, these plants were not documented before at the VOP, most likely due to their absence or lack of popularity during the time frame of the previous survey [1]. Furthermore, differences in plant inventory, especially the higher proportion of introduced species in our study compared to Van den Berg’s 1960–1980’s study [1], may be influenced by environmental factors. Forest degradation and deforestation [65] caused by logging [66], cattle ranching [67], and fires [68] prompt a reduction in the availability of medicinal and native hardwood species, pushing a demand for introduced medicinal species to treat human diseases [19]. However, the most popular medicinal species in Belém listed by Shanley and Luz [19] partly overlap with our survey, but also partly with Van den Berg [1]. This implies that it is yet unknown what the impacts of deforestation are on the availability of medicinal plants at the VOP.

Van den Berg [1] initiated her market study in 1965, and it continued for two decades over a time span in which there was more intact rainforest habitat compared to the present [69]. However, our shorter study resulted in a more diverse medicinal plant species composition. We attribute this variation to our different methodological approaches due to our differing aims: while her aim was to document the most frequently occurring species, our aim was to find out the overall medicinal species diversity of the market. Because of these different approaches, a direct comparison of the species composition between studies remains problematic. However, this is inherent to studying ethnobotanical inventories over time and space [14, 15]. When comparing such historical ethnobotanical data, the only manner to overcome disparities in historical survey quality across studies is to clearly acknowledge differences in methodology. Our study highlights the importance of repeating market surveys, the necessity of establishing standard methodologies, and the exact documentation of the applied methodologies to ensure comparable results in future studies [10].

### Plant names preserved

Of the species our survey shared with Van den Berg [1], the majority of vernacular names overlapped, indicating that there has been little change in names over three decades at the VOP. The small proportion that did not overlap could, again, be related to shifts in health perceptions and the occurrence of diseases over time. For example, in our study *Cissus verticillata* was called, among other names, *insulina*, which could be due to a higher occurrence of diabetes in Belém than at the time of Van den Berg’s study [1, 64]. However, such differences in associated names were minor. This is in line with our expectations, as plant names tend to present a remarkable continuity over time, even for centuries, as was previously found in Brazil [14] and neighboring Suriname [22]. For example, *Acmella oleracea* was named *jambu* in our survey, and we traced back this name to circa 375 years ago in the *Historia Naturalis Brasiliae* [14], where it was documented for the same species, in a slightly different spelling (*nhambí* and *nhambu*).

Substantial transformations in social and environmental factors are needed for plant names to change. Migration plays a large role, as migrants usually bring along their own language, plant uses, and cultural elements, including words for plants that may later be included in the dominant language [14, 17, 26]. In Tanzania, vernacular plant names at urban markets changed as a result of the migration from rural to urban centers of people with different cultural and lingual backgrounds [17]. Apparently, Belém’s cultural and linguistic context and its natural surroundings have not changed enough in the past decades to stimulate large shifts in vernacular plant names. Even though Belém is a large and expanding city, its surroundings are still inhabited by peoples who have valued and traded in these herbal medicines in the past decades. Their ethnobotanical knowledge of medicinal plants, including vernacular names, is an essential element to their survival and a source of income. Thus, as people worked with these medicinal plants consistently over the past decades, and languages remained the same, it resulted in the preservation of these vernacular names.

Regarding presently recorded names of species, of which the species were not recorded previously at VOP, a broader analysis of names, involving ethnobotanical surveys in and around Pará, could further confirm or refute our results on long-term preservation of plant names in the area.

### Language origins

The largest part of the vernacular plant names at this Amazonian market bore names of Portuguese origin. For example, the vernacular name *arruda* (*Ruta graveolens*) is of Portuguese origin, and the species is native to the Mediterranean region where it is embedded in the local plant pharmacopeia and also used against the “evil eye” [70]. We noticed this that several vendors at the VOP wore a sprig of *arruda* to protect against the “evil eye,” an interesting usage also commonly found in Bahia among Candomblé practitioners [25]. Bussmann et al. [13] also found that most plants sold at markets in Colombia, Bolivia, and Peru have mostly Spanish names. However, in northern Peru, Bussmann and Sharon [12] found that Spanish names were mainly used for introduced and coastal plant species whereas plants from the montane forests were often referred to by their indigenous name. In our case, there were relatively few plants of European origin at the VOP market and most plants with Portuguese vernacular names were native Brazilian species, such as, *Mansoa alliacea*, called *cipó de alho* (garlic vine).

The second largest group of vernacular names had a Tupi origin. Names like *cajú*, *tapereba*, *buçu*, *sicuriju*, *jambu*, and *tauari* reflect an acquisition of indigenous words in the Brazilian lexicon for living organisms found in the natural world, also evidenced by the native and especially Amazonian plants that were sold at the market. Portuguese-speaking people have inhabited the Belém area since the seventeenth century, and their language gradually became the official language [71]. Nevertheless, Tupinambá indigenous people were still present around Belém during the turn of the twentieth century [72], and Tupi, Tupinambá, and *língua geral* (a mix of Tupi and Portuguese which served as lingua franca in the region) were spoken in Pará up until the nineteenth or twentieth centuries [73], alongside various other spoken languages also belonging to the Tupi-Guarani language family [74]. This indicates that while the Portuguese-speaking peoples that inhabited the area mainly used Portuguese words to describe the natural world, they also relied upon indigenous Tupi names to some degree. This indigenous influence is not only reflected in the vernacular plant names at the VOP and the number of native species, but also in medicinal and religious plant uses. For example, the thin papery inner bark of *Couratari guianensis* (*tauari*), whose presence in Belém and surroundings was not previously documented, is used during Amazonian indigenous and Afro-Brazilian ceremonies as tobacco paper to roll ritual cigars [75]. We also found combinations of Tupi and Portuguese names, such as *pião-paje* (*Jatropha podagrica* Hook.) and *jurema preta* (*Mimosa tenuiflora* (Willd.) Poir.). These are prime examples of names shaped by cultural exchange among people from different cultural and linguistic backgrounds in the area.

We found a few vernacular names that had sub-Saharan African origins. Two of these directly refer to Africa: *alecrim-de-Angola* (*Vitex agnus-castus*), native to the Mediterranean region, and *manjerona-de-Angola* (*Lippia thymoides*), native to Brazil. The term Angola comes originally from Kimbundu, a Bantu language spoken in Angola, and it etymologically refers to a country of people from the Bantu linguistic group [76]. This word was used in the past by European traders to indicate the western coastline of Central Africa [77]. Although it is unclear when *alecrim-de-Angola* entered into Portuguese vocabulary, *V. agnus-castus* is used in Afro-Brazilian ceremonies in baths, as in the *Banho de São João* (bath of Saint John), an Afro-Brazilian ritual during the commemoration of St. John in Belém [9].

Further, the vernacular name *manjerona-de-Angola* has been documented before in Belém, but associated with *Origanum majorana* L. instead of *Lippia thymoides* [9]. A closely related plant, *Lippia multiflora* Moldenke, is used as medicine and in rituals along the western coast of Africa [78, 79]. We suggest that *L. multiflora* was replaced in use by *L. thymoides* in Belém, and it is possibly used in Afro-Brazilian ceremonies.

The last vernacular name with an African etymology, *tapete de Oxalá* (rug of Oxalá in English, *Episcia cupreata*), is also used in other parts of Brazil for other plant species [25]. Oxalá is a Candomblé deity and has a West African etymology, but it is unknown when and how this name was introduced into the Brazilian Portuguese vocabulary [24], and therefore how and by whom *Episcia cupreata* was first associated with this deity.

Thus, contrary to our expectation, we found very few vernacular names of medicinal and ritual plants sold at the Ver-o-Peso with African-derived origins. Yet, many of the plants we documented at the VOP with Portuguese and Tupi vernacular names, including African and non-African species, are used in rituals and ceremonies performed by followers of Afro-Brazilian religions, like Candomblé [1, 9, 25, 80, 81]. Other areas in Brazil, for instance in the northeast, where the Afro-Brazilian population is larger and may have experienced less difficulty in preserving Yoruba and Kikongo language elements, may exhibit other vernacular plant name patterns, including more African-derived plant names [25]. So, while we documented only a few plant names of African origin, several plants sold at the VOP were associated with ritual uses that were influenced by Afro-Brazilians. These findings may give us a glance into history: enslaved Africans who were brought to Brazil and arrived in the area of Belém, and later Afro-Brazilians faced more difficulties in retaining their language, possibly because they were in fewer numbers than Portuguese and Tupi-speaking peoples. However, they were clearly able to keep their knowledge on plant uses alive and to adjust their ceremonial and ritualistic customs to the new plants encountered in Brazil.

## Conclusions

A great variety of medicinal plant species, mainly of Amazonian origin, is sold at the medicinal plant stalls of the VOP. Just 30 of the 126 plant species we encountered overlapped with Van den Berg’s earlier market survey [1], and we reported a larger number of species and vernacular names not previously recorded at VOP. Also, there was a greater number of introduced species than in 1984 (Van den Berg); however, if this is due to changed environmental factors or to differences in methodologies remains unclear. In the case of vernacular names, we found that vernacular names of plants did not change much in the last three decades. Furthermore, the diverse origin of vernacular plant names reflects how the need for words to describe natural phenomena in the colonized and foreign land impelled Portuguese-speaking peoples to use their own reference words to name native Brazilian plants, but not without taking up several Tupi plant names. Although we only found a few plants with African-derived names, African heritage was not that much embedded in terms of language, but in associated ceremonial uses. In summary, the species sold at the VOP and their vernacular names, as well as the uses associated with these species found in literature, attest to the fact that Belém harbors an intricate syncretism of ethnobotanical knowledge of indigenous, Afro-Brazilian and European origin.

## Supplementary Information

**Additional file 1.** Geographic distribution and current status in Brazil. Description: Additional information on the geographic distribution and current status of medicinal and ritual plants found at the Ver-o-Peso in 2018.

## Data Availability

The datasets used during the current study are included in this published article and its additional information files. Voucher specimens of plants are stored in the herbarium of Museu Paraense Emílio Goeldi. Photos of plants are available from the first author on request.

## Abbreviations

VOP: The Ver-o-Peso market
MG: Herbarium of the Museu Paraense Emilio Goeldi
L: Herbarium of Naturalis Biodiversity Center
GBIF: Global Biodiversity Information Facility website [24]
